# French validation of the Compensatory Eating and Behaviors in Response to Alcohol Consumption Scale (CEBRACS) in a university student sample

**DOI:** 10.1007/s40519-023-01622-8

**Published:** 2023-11-10

**Authors:** Ludivine Ritz, Nicolas Mauny, Pascale Leconte, Nicolas Margas

**Affiliations:** 1 UNICAEN, Laboratoire de Psychologie Caen Normandie (LPCN, UR 7452), Pôle Santé, Maladies, Handicaps-MRSH (USR 3486, CNRS-UNICAEN), Normandie Univ, 14000 Caen, France; 2 UMR-S 1075 Inserm/Unicaen COMETE-Mobilités: Vieillissement, Pathologies, Santé, Caen, France; 3 Institut Des Sciences du Sport, Université de Lausanne, Lausanne, Switzerland; 4 UFR de Psychologie, Bâtiment L, Esplanade de La Paix, 14032 Caen Cedex 5, France

**Keywords:** Food, Drunkorexia, Alcohol, Eating disorders, Scale validation

## Abstract

**Purpose:**

Food and Alcohol Disturbance (FAD) is characterized by the combination of problematic alcohol use and eating disorder symptoms to offset caloric intake associated with alcohol drinking and/or to enhance intoxication. The Compensatory Eating and Behaviors in Response to Alcohol Consumption Scale (CEBRACS) is a proven tool for measuring FAD, validated in English and Italian populations but never in the French population. The present study aims at validating a French version of the CEBRACS in a representative sample of university students and to determine its validity and reliability.

**Methods:**

2267 university students completed the CEBRACS and measures of eating disorders, alcohol consumption and exercise.

**Results:**

An exploratory factor analysis revealed a 4-factor structure: enhancement of the effects of alcohol, dietary restraint and exercise, purging and vomiting and extreme fasting. The internal consistency for these subscales ranged from good to excellent. Correlations between the CEBRACS and eating disorders, alcohol and exercise measures revealed a good concurrent validity. No gender differences were found in the CEBRACS scores. Participants with a CEBRACS total score > 21 points were at higher risk for developing eating disorders and alcohol-related problems.

**Conclusions:**

These findings highlight the reliability and validity of the French version of the CEBRACS. The distinct factors identified in the CEBRACS allow to distinguish between participants with different motives for engaging FAD behaviour and thus to prevent future development of eating and/or alcohol use disorders. The CEBRACS seems to be a relevant scale to capture FAD behaviors and thus to prevent negative and deleterious consequences.

*Level of evidence*: Level III, evidence obtained from well-designed cohort or case–control analytic studies.

**Supplementary Information:**

The online version contains supplementary material available at 10.1007/s40519-023-01622-8.

## Introduction

Alcohol is the most widely used substance among young adult and students [1] and frequently co-occurs with eating disorders, even in nonclinical samples [2, 3]. This combination of alcohol use and eating disorders was first described in 2008 by Kershaw [4] and called drunkorexia. However, this term does not totally capture the phenomenon for which it is intended. This is why, more recently, the term “Food and Alcohol Disturbance” (FAD, [5]) has been coined to describe a set of eating disorders behaviors occurring before, during or after alcohol use, which aims to compensate for alcohol-related calories intake and/or to maximize the psychoactive effects of alcohol. A growing body of literature argues that between 6% and 39% of young adult reported reducing calories intake before drinking [3, 6–8] and that up to more than 50% of college students present FAD [9, 10]. Some studies have shown that students who experienced FAD reported more alcohol-related problems [11] and were more likely to report memory loss, sexual assault, injuries or fights [12]. Thus, FAD is a particularly harmful health phenomenon and its assessment is essential to better understand this disorder and develop preventive actions. Although various measures exist to evaluate FAD behaviors in students (for review see [11]), the majority of research used the Compensatory Eating and Behaviors in Response to Alcohol Consumption Scale (CEBRACS) [13]. The CEBRACS tends to lead to more comprehensive findings in terms of the conceptualization and operational criteria of FAD behaviors [11]. The advantage of this scale is that it includes items assessing both the use of behaviors to enhance the psychoactive effects of alcohol, and caloric compensation to avoid alcohol-related weight gain. In addition, the CEBRACS evaluates the frequency of a wide range of compensatory behaviors (dietary restraint, use of laxative and diuretics, exercising, extreme fasting, vomiting), and motives (getting drunk more, feeling the effects of alcohol faster, compensating for calories) at different times (before participants plan to drink alcohol, while drinking or under the effects of alcohol, and after drinking alcohol when no longer under the effects of alcohol).

The initial version of the 21-items CEBRACS was validated on 274 American college students [13]. The factor analysis conducted together with a principal component analysis (PCA) revealed 4 factors: alcohol effects, bulimia, dieting and exercise and restriction. The results showed an excellent internal consistency (ranged from 0.79 to 0.95) and a good concurrent validity with correlations between the CEBRACS factors and measures of alcohol consumption and severity of eating disorders symptomatology, including bulimia symptoms, body dissatisfaction, restriction and drive for thinness. The psychometric properties (factorial structure, internal consistency and concurrent validity) of the Italian version of the CEBRACS tested on 640 students showed a 5-factor structure, including the enhancement of the effects of alcohol, laxative use, dietary restraint and exercise, diuretic use and restriction and vomiting, suggesting a satisfactory construct validity [14]. As for the initial version, the internal consistency of the scale was good to excellent (ranging from 0.71 to 0.92) and the correlations observed between the CEBRACS factors and both eating disorders symptoms (body dissatisfaction, drive for thinness and bulimia symptoms) and alcohol-related issues suggest good concurrent validity. Additional analyses have shown an excellent degree of reproducibility (ICC = 0.806) for test–retest reliability [14].

However, subsequent studies [15, 16] conducted among American students failed to replicate the internal structure found in the initial English version of the CEBRACS [13]. Nevertheless, despite a relatively poor value of the comparative fit index (CFI = 0.85) resulting from the factor structure tested by [15] or root mean square error of approximation (RMSEA = 0.105) in [16], the other statistical parameters of the internal structure of the CEBRACS were good and the excellent and internal consistency of the scale was repeatedly ensured in all of the studies, even in those conducted in adults [7] and non-student populations [17]. Yet, the stability of the CEBRACS should not be questioned considering that (1) discrepancies in the internal structure of the CEBRACS in these previous studies may be the result of the selection sampling (students vs undergraduate students in Psychology vs teenagers), (2) some studies have been conducted in a relatively small sample of participants (respectively, *N* = 586 and *N* = 582), and (3) these discrepancies may be due to inter-cultural differences in alcohol consumption severity. The CEBRACS has been validated in English [13] and in Italian [14] but, not yet in French.

### Overview of the present study

Given the increasing prevalence of FAD among French university students (up to 56%); [9] and the co-occurrence of the risk of developing alcohol use and eating disorders [18], the aim of the present study was to validate a French version of the CEBRACS in a representative sample of university students and to determine its validity and reliability. The first sept of this work was to translate the initial English version of the CEBRACS based on a double-blind approach: from English to French and from French to English. Considering the heterogeneity of the internal factor structure of the CEBRACS, the internal structure of the French version of the CEBRACS administered in a large university sample will be tested by an exploratory factor analysis (EFA). Furthermore, regarding the findings of the previous studies that had used the CEBRACS, it was expected to find several subscales in the factor structure in our sample of university students. The internal consistency has been examined for the total CEBRACS and for the subscales resulting from the EFA. The convergent validity of the CEBRACS has been examined with measures, including alcohol consumption, eating disorders and exercise as well as comparisons between men and women. As the CEBRACS was found to reflect both caloric restriction and the psychoactive effects of alcohol, it was expected that the factorial subscales would be associated with the variables relating to alcohol and eating disorders. Since the gender differences are inconsistent across studies and that women are over-represented [11, 13, 14, 19–21], men and women were compared based on the measures of the CEBRACS, alcohol and eating disorders. Finally, the validity of a total cutoff score on the CEBRACS was tested.

## Materials and methods

### Participants

**Fig. 1:**
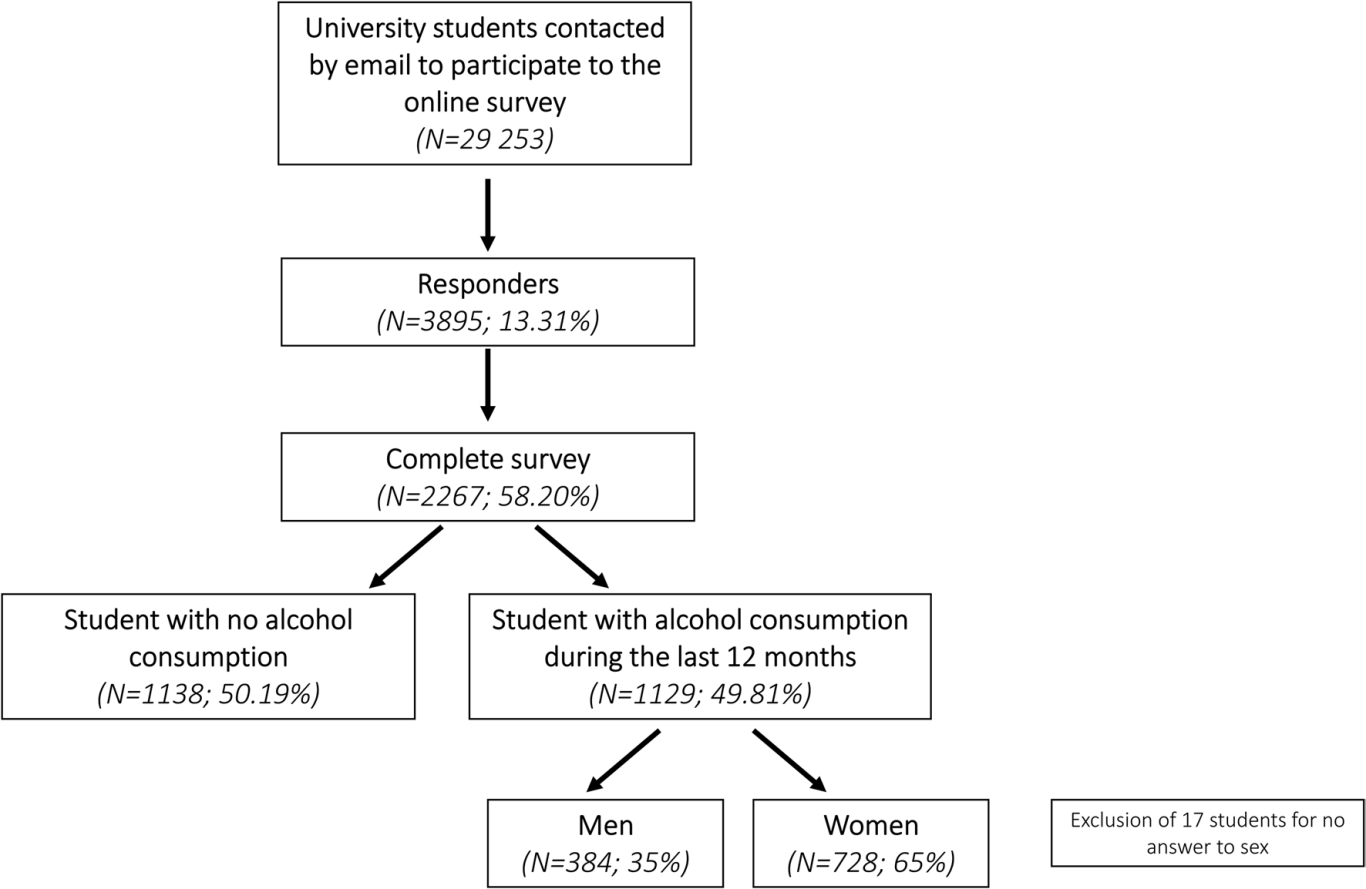
Flowchart of inclusion process

This study is part of a larger research program devoted to examining substance consumption among young adults (ADUC; Alcohol and Drugs at University of Caen). A sample of 2267 students was recruited at the University of Caen Normandy (France) through an online survey (November 2021) developed with the help of the Limesurvey® application and hosted by the server of the University of Caen (see Fig. 1 for data inclusion process). The response rate (13%) and ratio between completed response and included participants (58%) were similar to those obtained in previous studies conducted in college students [22, 23]. All of the participants were native French speakers. Among the 2267 students who completed the survey, 1129 (50%) reported alcohol consumption in the last 12 months. Seventeen students were withdrawn from the study for having failed to answer the gender question. Thus, 1112 participants made up the final sample and were included in the subsequent statistical analyses (see Table 1 for characteristics of the participants).

**Table 1:** Sample characteristics of the participants (*N* = 1112)

Socio-demographic variables	
Age	20.3 ± 2.59
Range	18–36
Gender (men/women)	384/728
Alcohol variables	
AUDIT	7.13 ± 5.37
Range	0–34
Age of onset	15.6 ± 1.71
Range	7–23
Number of standard drinks per week	4.84 ± 8.07
Range	0–100
Number of day per week of alcohol consumption	1.77 ± 1.13
Range	1–7
Eating disorders variables	
SCOFF	0.83 ± 1.08
Range	1–5
Frequency of laxatives/diuretic uses	0.03 ± 0.24
Range	0–4
Frequency of dietary restraint	0.58 ± 1.17
Range	0–4
Frequency of exercising	0.33 ± 0.95
Range	0–4
Food and alcohol disturbance	
CEBRACS	23.5 ± 6.17
Range	21–76

Except for gender, data are shown as mean ± standard deviation

*AUDIT* Alcohol Use Disorders Identification Test, *CEBRACS* Compensatory Eating and Behaviours in Response to Alcohol Consumption Scale

### Ethics

The study was notified and authorized by the “National Commission for Information Technology and Civil Liberties” with the number u24-20171109-01R1. Since the students were invited to participate via their formal university e-mail address, the University Information System Direction has developed a security system guaranteeing complete anonymity to the responders. All participants were provided with information on the subject matter of the study (purpose of the study and data collection) prior to their inclusion and gave their written informed consents, in accordance with the Declaration of Helsinki [24]. The Ethical Principles of Psychologists and Code of Conduct of the American Psychological Association [25] for the ethical treatment of human participants were respected for all the participants.

### Measures

#### Food and alcohol disturbances: the Compensatory Eating and Behaviors in Response to Alcohol Consumption Scale (CEBRACS)

First, the English version of the CEBRACS, validated by Rahal et al. [13], was translated into French by two people: an English native speaker student of the University of Caen and a psychologist with expertise in the field of eating disorders. Second, the translated French version was re-translated into English by another English native speaker student blind to the original version. Third, the same student compared the two English versions to determine the high degree of similarity between the English translation and the original version (see Supplementary Material for the French version of the CEBRACS). The CEBRACS is a 21 items Likert scale. The participants had to rate items depending on the frequency of the behavior [1 = never; 2 = rarely (approximately 25% by occasions); 3 = sometimes (approximately 50% by occasions); 4 = often (approximately 75% by occasions); 5 = nearly always] for three time periods: before drinking, while under the effects of alcohol (while drinking), and after the effects of alcohol have worn off (after drinking). Each of the three time periods assesses the same compensatory behaviors with items, including eating less than usual, skipping meals or entire day of eating, eating low-fat or low-calorie food, exercising, vomiting, and using diuretics or laxatives. The total score ranges from 21 to 105 points.

#### Alcohol variables

Alcohol consumption was assessed using the French version of the Alcohol Use Disorders Identification Test (AUDIT; [26]). AUDIT is a 10-item questionnaire designed to identify individuals at risk of developing alcohol-related problems. The AUDIT has been validated in French and recommended as an effective alcohol measure to be used in college students [27]. An AUDIT score ≥ 6 for women and ≥ 7 for men reflects a risk for alcohol-related problems [26]. Students were also asked about the age at which they first drank alcohol, their level of alcohol consumption per week (in standard drinks, a standard drink corresponding to a beverage containing about 10 g of pure alcohol) and their frequency of drinking during a typical week (ranging from 1 to 7 days).

#### Eating disorders

The SCOFF questionnaire is designed to screen the risk of eating disorders in at-risk populations and student populations [28, 29]. The SCOFF questionnaire is composed of five dichotomous questions (“yes” or “no” answers) and scored from 0 to 5 according to the sum of the score of each of the five answers. The established threshold is of at least two positive answers. The SCOFF sensitivity and specificity were 94.6% and 94.8%, respectively, for eating disorders in the student population [28]. Students were also asked about how frequently they used laxative in the past 3 months (from never “0” to very often “4”), how frequently they limited food intake at each meal (dietary restraint) in the past 3 months (from 0 never to 4 very often) and how frequently they practice a physical activity to burn calories (from never “0” to very often “4”).

### Statistical analyses

The normality of the distribution was examined with the Shapiro–Wilk test and both skewness and kurtosis parameters. The factor structure of the French version of the CEBRACS was determined by a factor analysis using EFA. The Promax method was used for the rotation procedure and bootstrap CI method with 1000 iterations. Only factors with an eigenvalue > 1 were extracted and considered as significant [30]. The Bartlett sphericity test was computed to test the hypothesis of identical matrix and conclude for the adequate structure of data for factor analysis. The reliability of the identified factors of the CEBRACS was test by Cronbach’s alpha coefficients to evaluate internal consistency. Value > 0.80 was considered as excellent.

Concurrent validity was determined by correlation analyses between each of the identified factors of the CEBRACS and both alcohol (scores of the AUDIT, age of onset, number of standard drinks per week, number of days per week of alcohol consumption) and eating disorders variables (SCOFF, frequency of laxatives/diuretic uses, frequency of dietary restraint and frequency of exercising). Comparisons between men and women on the CEBRACS, alcohol and eating disorders variables were carried out. Bonferroni correction was applied to prevent type I error.

## Results

Shapiro–Wilk tests conducted on the 21 items of the CEBRACS and analysis of skewness and kurtosis parameters showed a violation of normality (respectively, *p* ≤ 0.001 and both skewness and kurtosis values > 1). A robust maximum likelihood estimator and non-parametric statistical tests were used in the subsequent analyses to take into account for data non-normally distributed.

### Factor analysis with EFA

**Fig. 2:**
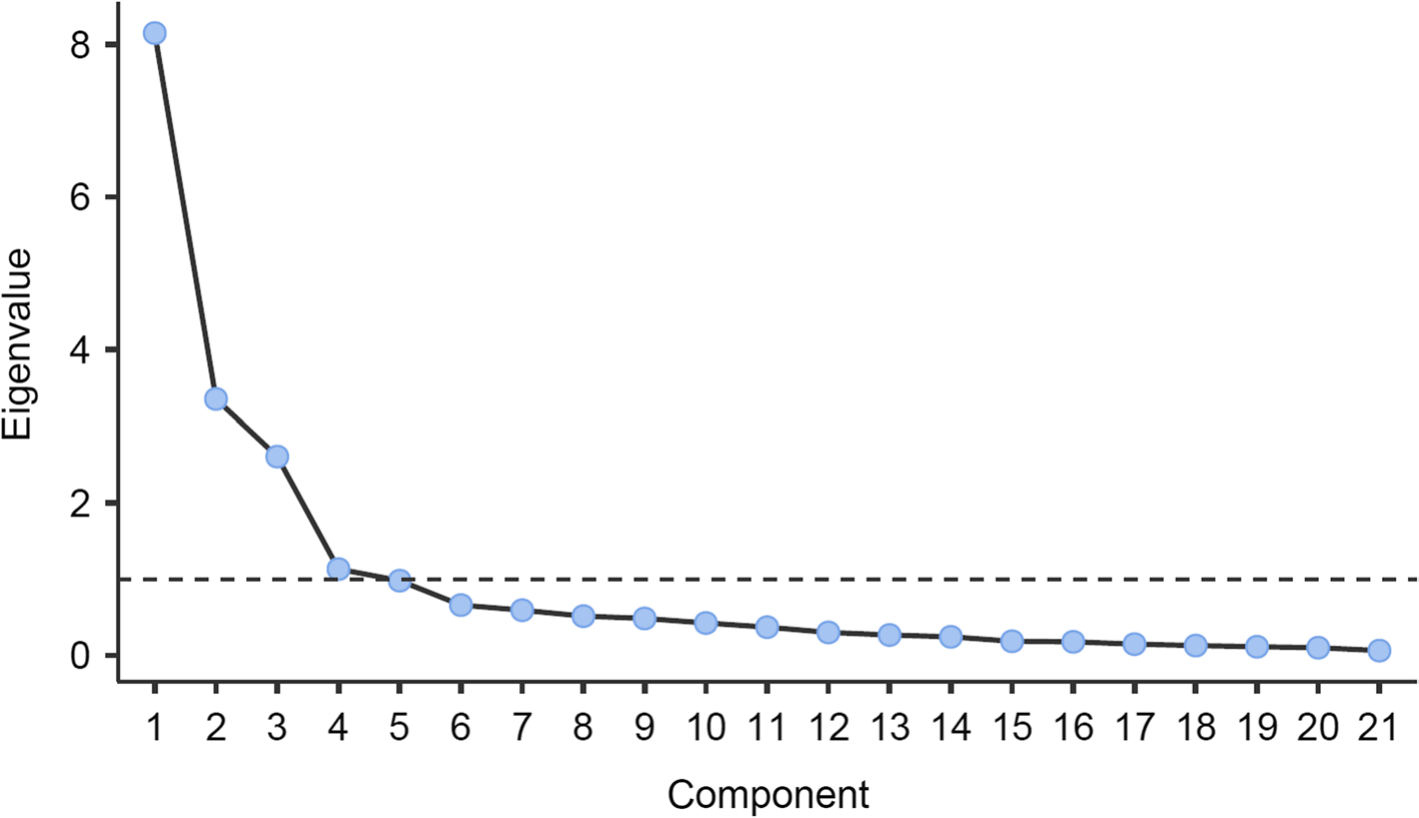
Scree plot resulting from the exploratory factor analysis (EFA)

The analysis of the scree plot (Fig. 2) resulting from the EFA conducted with bootstrap (1000 iterations) and eigenvalues set at > 1 indicated that a 4-factor solution was the best fit to the data (Table 2). Factor 1 contained 7 items reflecting behaviors to enhance the effect of alcohol to get drunk faster. Factor 2 contained 7 items reflecting dietary restraint and exercise. Factor 3 contained 5 items and appeared to reflect purging behaviors. Factor 4 contained 2 items (vomiting and skipping an entire day of eating) and seemed to reflect vomiting and extreme fasting. The Bartlett sphericity test (Chi-square: 20,186; *p* < 0.001) allowed to reject the hypothesis of identical matrix, concluding that data had an adequate structure for factoring. The overall Kaiser–Meyer–Olkin (KMO) measure of sampling value was 0.890, indicating that the sampling was adequate for factor analysis [31].

**Table 2:** Factor structure derived from the exploratory factor analysis (EFA) of the French version of the CEBRACS

CEBRACS items		Component loadings			
		Factor 1	Factor 2	Factor 3	Factor 4
CEBRACS 1	Before—eating less to get drunker	0.864			
CEBRACS 3	Before—eating less to get drunk faster	0.940			
CEBRACS 6	Before—skipping meals to get drunk faster	0.853			
CEBRACS 7	During—eating less to get drunk faster	0.934			
CEBRACS 9	During—not eating to get drunk faster	0.884			
CEBRACS 12	During—eating less to get drunker	0.954			
CEBRACS 14	During—not eating to get drunker	0.896			
CEBRACS 2	Before—exercising		0.952		
CEBRACS 4	Before—skipping meals		0.473		
CEBRACS 10	During—eating low calorie food		0.507		
CEBRACS 11	During—drinking low caloric alcoholic drink		0.527		
CEBRACS 16	After—eating low calorie food		0.681		
CEBRACS 18	After—exercising		0.999		
CEBRACS 20	After—eating less		0.600		
CEBRACS 5	Before—laxative use			0.675	
CEBRACS 8	During—diuretic use			0.732	
CEBRACS 13	During—laxative use			0.894	
CEBRACS 15	After—diuretic use			0.940	
CEBRACS 17	After—laxative use			0.921	
CEBRACS 19	After—vomiting				0.928
CEBRACS 21	After—skipping an entire day of eating				0.857

### Internal consistency

The Cronbach’s alpha for the overall CEBRACS was 0.912, 0.944 for Factor 1, 0.885 for Factor 2, 0.864 for Factor 3 and 0.680 for Factor 4.

### Concurrent validity

Spearman correlations between each factor and the total score of the CEBRACS and the alcohol and eating disorders variables are presented in Table 3. Only Spearman’s rho with Bonferonni correction (*p* ≤ 0.001 for 40 correlations) and a medium effect size (> 0.20) were considered as significant. Factor 1, reflecting the “enhancement of the effects of alcohol” was significantly correlated with the AUDIT score, the number of standard drinks per week, the SCOFF score and the frequency of dietary restraint. Factor 2 (“Dietary restraint and exercise”) was significantly correlated with the SCOFF score, the frequency of dietary restraint and the frequency of exercising. Factor 3 (“Purging”) was correlated with the frequency of laxative and diuretic uses. Factor 4 (“Vomiting and extreme fasting”) was correlated with the SCOFF score and the frequency of dietary restraint. The total score of the CEBRACS was correlated with the AUDIT total score, the SCOFF score, the frequency of dietary restraint and the frequency of exercising.

**Table 3:** Results of the correlation analyses conducted between the four factors and the total score of the CEBRACS and alcohol and eating disorders variables

	CEBRACS Factors				Total score
	Factor 1	Factor 2	Factor 3	Factor 4	
	“Enhancement the effects of alcohol”	“Dietary restraint and exercise”	“Purging”	“Vomiting and extreme fasting”	
Alcohol variables					
AUDIT	0.31^***^	0.17^***^	0.04	0.18^***^	0.29^***^
Age of onset	− 0.12^***^	− 0.05	− 0.03	− 0.11^***^	− 0.10^***^
Number of standard drinks per week	0.18^***^	0.08^*^	− 0.01	0.09^**^	0.14^***^
Number of day per week of alcohol consumption	0.14^***^	0.08^**^	0.03	0.12^***^	0.11^***^
Eating disorders variables					
SCOFF	0.23^***^	0.25^***^	0.04	0.25^***^	0.28^***^
Frequency of laxatives/diuretic uses	0.12^***^	0.12^***^	0.13^***^	0.15^***^	0.12^***^
Frequency of dietary restraint	0.19^***^	0.29^***^	0.08^**^	0.22^***^	0.28^***^
Frequency of exercising	0.15^***^	0.29^***^	0.04	0.15^***^	0.25^***^

Data are shown as rho of Spearman

^***^p ≤ 0.001; ^**^p ≤ 0.01; ^*^p ≤ 0.05; Data in bold reflect medium effect size (> .20)

### Comparisons between men and women on the CEBRACS, alcohol and eating disorders variables

The results of the Mann–Whitney tests that compared men and women on the CEBRACS, alcohol and eating disorders variables are presented in Table 4. After Bonferonni correction, the SCOFF score and the frequency of dietary restraint were higher in women than in men. The number of standard drinks per week, the number of days of drinking per week and the AUDIT score were higher in men than in women. No significant differences were found between men and women on the subscales of the CEBRACS and the CEBRACS total score.

**Table 4:** Comparisons between men and women of our sample on the alcohol, eating disorders and CEBRACS variables

Variables	Men (*N* = 384)	Women (*N* = 728)	*p* value
Alcohol variables			
AUDIT	8.39 ± 6.08	6.46 ± 4.83	0.001*
Age of onset	15.41 ± 1.86	15.75 ± 1.62	0.009
Number of standard drinks per week	7.11 ± 11.24	3.62 ± 5.30	0.001*
Number of day per week of alcohol consumption	2.03 ± 1.34	1.63 ± 0.97	0.001*
Eating disorders variables			
SCOFF	0.48 ± 0.80	1.00 ± 1.16	0.001*
Frequency of laxatives/diuretic uses	0.00 ± 0.05	0.04 ± 0.29	0.020
Frequency of dietary restraint	0.36 ± 0.96	0.69 ± 1.26	0.001
Frequency of exercising	0.34 ± 0.97	0.32 ± 0.93	0.800
CEBRACS			
Total score	22.76 ± 4.87	23.87 ± 6.72	0.008
Factor 1	7.93 ± 3.16	8.33 ± 3.61	0.010
Factor 2	7.73 ± 2.07	8.33 ± 3.61	0.080
Factor 3	5.04 ± 0.57	5.05 ± 0.61	0.610
Factor 4	2.06 ± 0.37	2.16 ± 0.79	0.040

Data are shown as mean ± standard deviation

*AUDIT* Alcohol Use Disorders Identification Test, *CEBRACS* Compensatory Eating and Behaviours in Response to Alcohol Consumption Scale

*Significant after Bonferonni correction (*p* ≤ 0.004 for 13 comparisons)

### Validity of a CEBRACS cutoff score

**Fig. 3:**
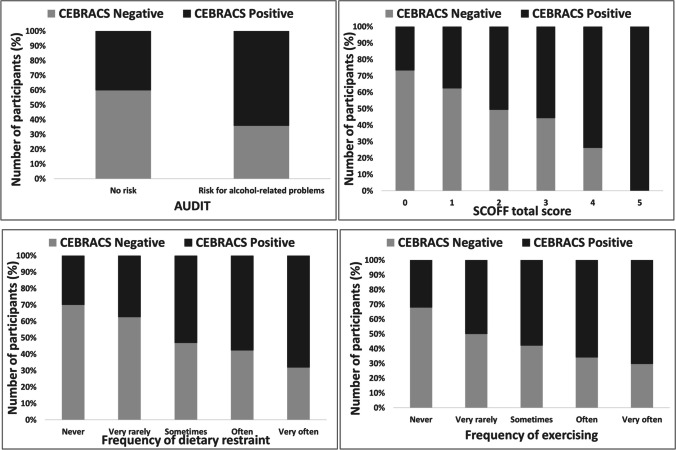
Proportion of participants (in %) on the alcohol and eating disorders variables, regarding the negative or positive CEBRACS score. Negative CEBRACS score = 21; Positive CEBRACS score > 21. At the top left: proportion of participants regarding the AUDIT score, with no risk of developing alcohol-related problems (AUDIT score < 6 for women and 7 for men) and risk of developing alcohol-related problems ≥ 6 for woman and ≥ 7 for men). At the top right: proportion of participants regarding the SCOFF score. A score ≥ 2 indicates a risk of developing eating disorders. At the bottom left: proportion of participants on the frequency of dietary restraint. At the bottom right: proportion of participants on the frequency of exercising

The participants were divided into two groups based on the CEBRACS total score: the participants with a positive CEBRACS score > 21 points (who declare that they rarely engage in FAD behavior (approximately 25% by occasions) at least on one item) and the participants with a negative CEBRACS score = 21 (who declare that they never engage in FAD behavior). These two groups were compared using the Mann–Whitney test for variables correlated with the CEBRACS total score (SCOFF score, AUDIT total score, frequency of dietary restraint and frequency of exercising). The results are presented in Table 5. All comparisons were statistically different, with participants with a positive CEBRACS score having higher scores on the SCOFF score, AUDIT score, frequency of dietary restraint and frequency of exercising than participants with a negative CEBRACS score. Figure 3 depicts the proportion of participants (in %) on the AUDIT score, SCOFF score, frequency of dietary restraint and frequency of exercising, regarding the positive or negative CEBRACS score. The percentage of participants with a positive CEBRACS score was higher (64%) among those being at higher risk of developing alcohol-related problems (i.e., AUDIT score ≥ 6 for women and ≥ 7 for men). Similarly, the percentage of participants with a positive CEBRACS score was higher (51%) among those being at higher risk of developing eating disorders (i.e., SCOFF score ≥ 2) and seemed to increase along with the severity of the SCOFF score (Fig. 3). Finally, the percentage of participants with a positive CEBRACS score was higher in those who reported sometimes dietary restrictive eating and exercise behaviors (53% and 58%, respectively) and seemed to increase along with the frequency.

**Table 5:** Comparisons between participants with negative CEBRACS total score (21 points) and participants with positive CEBRACS total score (> 21 points) on the alcohol and eating disorders variables

Variables	CEBRACS		Statistics
	Negative score (= 21 points) *N* = 714	Positive score (> 21 points) *N* = 398	
AUDIT	6.07 ± 0.37	9.02 ± 0.31	*U*_(1110)_ = 96,034; *p* < 0.001*; *r* = 0.32
Range	0–30	1–34	
SCOFF	0.62 ± 0.93	1.18 ± 1.23	*U*_(1110)_ = 105,274; *p* < 0.001*; *r* = 0.26
Range	0–4	0–5	
Frequency of dietary restraint	0.37 ± 0.95	0.95 ± 1.41	*U*_(1110)_ = 113,094; *p* < 0.001*; *r* = 0.20
Range	0–4	0–4	
Frequency of exercising	0.19 ± 0.73	0.59 ± 1.21	*U*_(1110)_ = 121,833; *p* < 0.001*; *r* = 0.14
Range	0–4	0–4	

Data are shown as mean ± standard deviation

*AUDIT* Alcohol Use Disorders Identification Test, *CEBRACS* Compensatory Eating and Behaviors in Response to Alcohol Consumption Scale, *U* of Mann–Whitney; *r* effect sizes were determined with rank biserial correlation (*r* < 0.10 small effect size; *r* < 0.30 medium; *r* < 0.50 large)

^*^Significant at *p* ≤ 0.05

## Discussion

The aim of the present study was to validate a French version of the CEBRACS in a representative large sample of students and to determine its validity and reliability in relation to alcohol consumption and eating disorders severity. The CEBRACS was developed to assess FAD behaviors with both alcohol effects and eating compensatory motivations at three different times (before, during and after alcohol consumption).

The findings of the EFA revealed four factors that were partially consistent with those of the English [13] and Italian versions [14] of the CEBRACS. The first factor identified in our study, termed «enhancement of alcohol effect», contains the same items and coincides fully with the same factor identified in previous studies [13, 14]. In our study, factor 2, which refers to “dietary restraint and exercise”, coincides with factor 3 named “Dietary restraint and exercise” in both studies [13, 14]. However, in our study, item 4 of the CEBRACS (before—skipping meals), is part of this factor and seems to be more relevant in this one than in the “Restriction” factor which rather reflects extreme restrictive behavior as described in [13]. Factor 3, considered as “Purging”, contains the same items as factor 2 “Bulimia” [13] and both factors 2 (“laxative use”) and 4 (“diuretic use) [14]. Factor 4, reflecting “Vomiting and extreme fasting”, partially overlaps factors 4 and 5, respectively, in the English and Italian versions of the CEBRACS [13, 14]. It incorporates items with severe behaviors (skipping an entire day of eating and self-vomiting) that are more relevant in this factor than items included in the original version and in the Italian version, reflecting more dietary restraint (before—skipping meals and after—eating less). The internal consistency of these four factors ranged from good to excellent.

The relative discrepancies between the internal structure of the CEBRACS reported in previous studies and that of our study are minor (only for the factor 4) and likely reflect a more severe engagement in extreme behaviors, such as self-vomiting and extreme fasting after drinking on the part of French students than on the part of American or Italian students [13, 14]. These inter-cultural differences in FAD behaviors have already been reported in a recent study [9]. In this study conducted among American and French students, although the prevalence of FAD behaviors was similar in both nationalities (about half of the participants), drive for thinness and alcohol consumption were culturally moderated. French students with high concerns of drive for thinness were more engaged in FAD behaviors for compensatory reasons, whereas American students with the higher levels of drinking problems were more likely to be engaged in FAD behaviors for compensatory purposes and to enhance the effects of alcohol. Overall, these findings suggest that although FAD is observed cross-culturally, the motive for the behavior varied depending on the culture and nationality. This point should be taken into consideration when assessing FAD behaviors.

The relationships found between the CEBRACS scores and measures of alcohol consumption, alcohol-related problems (AUDIT) and eating disorders (SCOFF, frequency of dietary restraint and exercising) have resulted in a good concurrent validity. More precisely, the CEBRACS factor “Enhance the effects of alcohol” is positively correlated with both alcohol and eating disorders variables, in agreement with previous studies [13, 19, 32]. This finding was not totally expected, given the participants’ motivation to restrict food mostly to enhance the intoxicating effects of alcohol, and given the findings of the study conducted by [14] in which the “alcohol effect” factor was not correlated with eating disorders symptomatology assessed with the Eating Disorder Inventory-3 (EDI-3). It may be explained by the fact that participants who engaged in FAD for motives related to alcohol effects are aware that their behaviors also reflect a dysfunctional eating pattern and that the combination of high alcohol consumption and extreme compensatory behaviors may have deleterious effects.

Conversely, the other factors highlighted in our study (“dietary restraint and exercise”, “purging” and “vomiting and extreme fasting”) were only associated with the eating disorders measures [13, 14, 19], suggesting that students who score high on these subscales are more likely to engage in maladaptive compensatory behaviors and suffer from eating disorders [18]. This also suggests that the primary motives of the participants who report engaging in this type of behavior are rather related to weight gain concerns.

The CEBRACS total score was related to the alcohol and eating disorders variables, as has been reported repeatedly in student [10, 19], adolescent [8] and adult populations [7] (for review see [11]. This result underlines the high risk for the participants to develop eating and alcohol use disorders. This also supports the conceptualization of FAD as being at the intersection between dysfunctional eating and drinking behavior.

Analyses of the gender effect failed to evidence significant differences between men and women on the CEBRACS total score and the four factors (with or without Bonferroni correction), as previously reported [13, 14, 17, 20, 21]. The literature has no consensus regarding gender differences on the CEBRACS (for review see [11]), probably due to the over-representation of the female population in the studies. Hence, the under-representation of men does not provide an adequate statistical comparison. However, although in our study women outnumbered men (65% vs 35%), distinct gender differences were found on drinking and eating patterns: women were more at risk of developing eating disorders, whereas men were more at risk of developing alcohol-related problems and have a higher level of alcohol consumption. The lack of these gender-related differences on the CEBRACS suggest that FAD may not just refer to the combination of drinking and eating disorders, but to a more complex behavior triggered by different motives and characterized by behavioral manifestations.

Finally, when the participants were categorized as having FAD behavior on the CEBRACS total score (> 21 points) and compared with those having no FAD behavior (CEBRACS total score = 21 points; i.e., never), significant differences were found between both groups in terms of the alcohol and eating disorders measures. FAD participants are more at risk of developing alcohol-related problems, eating disorders and have a higher level of alcohol consumption than the participants who never engage in this behavior. These findings are consistent with previous studies conducted in student [33] and adolescent populations [8]. A CEBRACS total cutoff score set at > 21 points, as used in several studies [7, 10, 13, 32] may be useful to identify students who engage in FAD behaviors and are at high risk of developing drinking and eating disorders.

### Strengths and limits

Our study includes some limitations, one of which was the relatively small sample of men, as previously pointed in precedent studies [13, 14]. Future studies should extend the validation of the CEBRACS to a larger proportion of men to form a sample that would be more representative. This study was only conducted in university students, while FAD behaviors were also reported in non-student populations [7, 17]. The assessment of FAD behaviors should also be conducted in a clinical sample suffering from eating and/or alcohol use disorders, to provide a global picture of this phenomenon and to allow the identification of comorbid factors. Finally, all variables were assessed using self-report measures, which is a possible factor of self-report bias (i.e., social desirability). However, given that students were contacted via email and completed an online survey, we expected a lower desirability bias.

The present study highlights the reliability and validity of the French version of the CEBRACS, with good internal consistency and concurrent validity. The strength of our study is that it included a large sample of university students with measures of alcohol consumption, alcohol-related problems and eating disorders. The distinct factors identified on the CEBRACS allow to distinguish participants with different motives for FAD engagement and thus contribute to prevent future development of eating and/or alcohol use disorders. A CEBRACS total score above 21 points may be a useful cutoff score for identifying students engaged in FAD behaviors, that can lead to significant health problems.

## Conclusion

Overall, these findings suggest that the CEBRACS is a relevant scale to capture FAD behaviors in French students, i.e., not only for purposes of avoiding weigh gain prior to alcohol consumption but also for the enhancement of the psychoactive effects of alcohol, with the use of restriction and purging strategies before, during and after alcohol drinking. Further studies are needed to specify distinct profiles of FAD participants in relation with psychological traits (depression, anxiety, impulsivity) and drinking motives (coping, conformity).

## What is already known on this subject

The prevalence of FAD among French university students is increasing and associated with the risk of developing alcohol use and/or eating disorders. Despite the excellent validity of the CEBRACS in Italian and English population to capture FAD behaviors, this proven scale was not validated in French population.

## What this study adds

The present study has validated a French version of the CEBRACS and provides a useful cutoff score for the identification of students engaged in FAD behaviors. The distinct factors identified on the CEBRACS allow to distinguish participants with different motives for FAD engagement and to prevent for a risk of future development of eating and/or alcohol use disorders.

### Supplementary Information

Below is the link to the electronic supplementary material.

Supplementary file1 (DOCX 16 KB)

## Data Availability

Data and measures are available: https://osf.io/9sytm/
