# Supplementary material for: French validation of the Compensatory Eating and Behaviors in Response to Alcohol Consumption Scale (CEBRACS) in a university student sample
Source: Eat Weight Disord. 2023 Nov 10;28(1):95. doi: 10.1007/s40519-023-01622-8 (PMC10638208; doi:10.1007/s40519-023-01622-8)
Supplement: Supplementary file 1 — Supplementary file1 (DOCX 16 KB) [file 40519_2023_1622_MOESM1_ESM.docx]

**Compensatory Eating Behaviors Related to Alcohol Consumption**

Pour les déclarations suivantes, réfléchissez aux comportements que vous avez adoptés AVANT de prévoir de boire de l'alcool. C'est-à-dire, pensez aux situations dans lesquelles vous saviez que vous alliez boire de l'alcool dans un futur proche (par exemple, vous avez prévu de sortir boire avec des amis, d’assister à un mariage ou à un anniversaire où vous prévoyiez de boire, ou d’assister à tout autre événement ou situation où vous saviez que vous alliez boire plus tard).

|  | Items | Réponses |
| --- | --- | --- |
| CEBRACS_1 | Au cours des trois derniers mois, j'ai mangé moins que d'habitude pendant un ou plusieurs repas avant de boire dans le but de m’enivrer. | Jamais  Rarement (environ 25% du temps)  Parfois (environ 50% du temps)  Souvent (environ 75% du temps)  Presque tout le temps |
| CEBRACS_2 | Au cours des trois derniers mois, j'ai fait de l'exercice avant de boire pour compenser les calories contenues dans l'alcool que j’avais prévu de consommer. |  |
| CEBRACS_3 | Au cours des trois derniers mois, j'ai mangé moins que d'habitude pendant un ou plusieurs repas avant de boire pour ressentir plus rapidement les effets de l'alcool. |  |
| CEBRACS_4 | Au cours des trois derniers mois, j'ai sauté un ou plusieurs repas avant de boire pour compenser le nombre de calories contenues dans l'alcool que j'avais prévu de consommer. |  |
| CEBRACS_5 | Au cours des trois derniers mois, j'ai pris des laxatifs avant de boire pour compenser les calories de l'alcool que j’avais prévu de consommer. |  |
| CEBRACS_6 | Au cours des trois derniers mois, j'ai sauté un ou plusieurs repas avant de boire pour ressentir plus rapidement les effets de l'alcool. |  |

Pour chacune des déclarations suivantes, réfléchissez aux comportements que vous avez adoptés PENDANT que vous buviez ou sous l'effet de l'alcool (par exemple, pendant que vous buviez lors d'une réception de mariage, d'une fête, dans un bar, un club, à un match de football). Cela inclut également les situations dans lesquelles vous avez peut-être cessé de boire à un moment donné, mais où les effets de l'alcool ne se sont pas complètement dissipés. Par exemple, imaginez que vous revenez d'une fête durant laquelle vous avez bu et que vous pouvez encore ressentir les effets de l'alcool même si vous avez arrêté de boire plus tôt dans la nuit.

| CEBRACS_7 | Au cours des trois derniers mois, j'ai mangé moins que d'habitude lorsque je buvais parce que je voulais ressentir les effets de l'alcool plus rapidement. | Jamais  Rarement (environ 25% du temps)  Parfois (environ 50% du temps)  Souvent (environ 75% du temps)  Presque tout le temps |
| --- | --- | --- |
| CEBRACS_8 | Au cours des trois derniers mois, j'ai pris des diurétiques pendant que je buvais pour compenser les calories de l'alcool que je consommais. |  |
| CEBRACS_9 | Au cours des trois derniers mois, je n'ai pas mangé du tout pendant que je buvais parce que je voulais ressentir les effets de l'alcool plus rapidement. |  |
| CEBRACS_10 | Au cours des trois derniers mois, j'ai mangé des aliments peu caloriques ou pauvres en graisses tout en buvant afin de compenser les calories de l'alcool que je consommais. |  |
| CEBRACS_11 | Au cours des trois derniers mois, j'ai bu de la bière ou des boissons alcoolisées à faible teneur en calories afin d’absorber moins de calories que celles contenues dans d’autres alcools. |  |
| CEBRACS_12 | Au cours des trois derniers mois, j'ai mangé moins que d'habitude pendant que je buvais parce que je voulais m’enivrer plus fortement. |  |
| CEBRACS_13 | Au cours des trois derniers mois, j'ai pris des laxatifs pendant que je buvais pour compenser les calories de l'alcool que je consommais. |  |
| CEBRACS_14 | Au cours des trois derniers mois, je n'ai pas mangé du tout pendant que je buvais parce que je voulais m’enivrer plus fortement. |  |

Pour chacune des déclarations suivantes, pensez aux comportements que vous avez adoptés APRÈS avoir bu de l'alcool et lorsque vous n’étiez plus sous l’influence de l’alcool. Il peut s'agir de votre comportement plus tard dans la journée, le lendemain ou plusieurs jours après que les effets de l'alcool se soient dissipés.

| CEBRACS_15 | Au cours des trois derniers mois, j'ai pris des diurétiques pour compenser les calories de l'alcool que j'avais consommé précédemment, lorsque j'étais sous l’influence de l'alcool. | Jamais  Rarement (environ 25% du temps)  Parfois (environ 50% du temps)  Souvent (environ 75% du temps)  Presque tout le temps |
| --- | --- | --- |
| CEBRACS_16 | Au cours des trois derniers mois, j'ai mangé des aliments peu caloriques ou pauvres en graisses pendant un ou plusieurs repas pour compenser les calories de l'alcool que j'avais consommé précédemment, alors que j'étais sous l’influence de l'alcool. |  |
| CEBRACS_17 | Au cours des trois derniers mois, j'ai pris des laxatifs pour compenser les calories de l'alcool que j'avais consommé précédemment, lorsque j'étais sous l’influence de l'alcool. |  |
| CEBRACS_18 | Au cours des trois derniers mois, j'ai fait de l'exercice pour compenser les calories contenues dans l'alcool que j'avais consommé précédemment, lorsque j'étais sous l’influence de l'alcool. |  |
| CEBRACS_19 | Au cours des trois derniers mois, je me suis fait vomir pour compenser les calories de l'alcool que j'avais consommé précédemment, alors que j'étais sous l’influence de l'alcool. |  |
| CEBRACS_20 | Au cours des trois derniers mois, j'ai mangé moins que d'habitude pendant un ou plusieurs repas pour compenser les calories contenues dans l'alcool que j'avais consommé précédemment, alors que j'étais sous l’influence de l'alcool. |  |
| CEBRACS_21 | Au cours des trois derniers mois, j'ai sauté une journée entière ou plus de repas pour compenser les calories de l'alcool que j'avais consommé précédemment, lorsque j'étais sous l’influence de l'alcool. |  |
